# Twisted Teeth: Ovarian Torsion Secondary to Mature Teratoma

**DOI:** 10.7759/cureus.6649

**Published:** 2020-01-14

**Authors:** Allen D Chang, Kiersten Carter

**Affiliations:** 1 Emergency Medicine, Stanford University Hospital, Palo Alto, USA; 2 Emergency Medicine, The Permanente Medical Group, Kaiser Permanente, Santa Clara, USA

**Keywords:** ovarian torsion, mature teratoma, pediatric abdominal pain, pediatric emergency medicine, pediatric ultrasound

## Abstract

Evaluation of undifferentiated pediatric abdominal pain presents a unique set of challenges, especially in the setting of inconclusive and limited diagnostic imaging. In this case report, a female child presented to the emergency department with persistent abdominal pain, normal lab studies, and unusual trans-abdominal pelvic ultrasound findings. Urgent exploratory laparoscopy was completed, demonstrating a torsed adnexa rotated around a very large, mature teratoma, with irregular masses consistent with fully developed teeth. Early recognition of atypical pediatric abdominal pain in the setting of equivocal diagnostic imaging findings and collaboration with surgical colleagues resulted in a positive outcome for this patient.

## Introduction

The approach to pediatric abdominal pain in the emergency department (ED) is often limited by both the patient’s participation in the assessment and a desire to be judicious with diagnostic testing and imaging. When ultrasound provides abnormal but inconclusive results, concern for ionizing radiation exposure can limit additional imaging options [1]. Ovarian torsion is a “cannot-miss” diagnosis for emergency medicine physicians, but this is often complicated by vague or intermittent symptoms as well as inherent limitations in diagnostic imaging. Children may also be poorer historians depending on their age group, which can also complicate the clinical picture. While a case series and literature review noted that more than 71% of ovarian torsion occurred in women older than 20 years, the mean age of pediatric cases was 12 years, likely related to a higher preponderance of ovarian cysts in menarchal or perimenarchal girls [2]. A high degree of clinical suspicion is necessary to appropriately engage surgical colleagues early for collaborative evaluation and optimize outcomes for these patients at high risk for long-term morbidity.

## Case presentation

An 8-year-old female who was previously healthy, fully vaccinated, with no history of abdominal surgery presented with an acute onset of periumbilical abdominal pain. The pain was described as constant in nature, non-radiating, and not associated with any recent falls or trauma. The mother reported one episode of non-bloody vomiting and denied associated urinary or bowel movement symptoms, atypical food/drink intake, recent travel history, or sick contacts. A thorough chart review did not demonstrate features concerning for abuse, and the patient was pre-menarchal.

On physical examination, the patient’s vital signs revealed mild hypertension (temperature of 98.6 F, blood pressure of 130/59 mm Hg, heart rate of 85 bpm, respiratory rate of 22 breaths/minute, 98% room air), and she was noted to be crying in bed and unable to lay comfortably in the gurney. Her abdomen was soft with normal bowel sounds, no distension, with left greater than right lower quadrant tenderness to palpation, voluntary guarding, and no rebound. Her external genital examination was unremarkable, with no bleeding or obvious evidence of trauma, and an internal examination was deferred due to patient discomfort and parental preference.

Emergency physicians were strongly concerned for ovarian torsion, especially with the mother reporting a similar presentation when she was a child. The team engaged surgical consultation with both gynecology and pediatric surgery departments early, who requested an appendix ultrasound in addition to the adnexal imaging already ordered.

**Figure 1:**
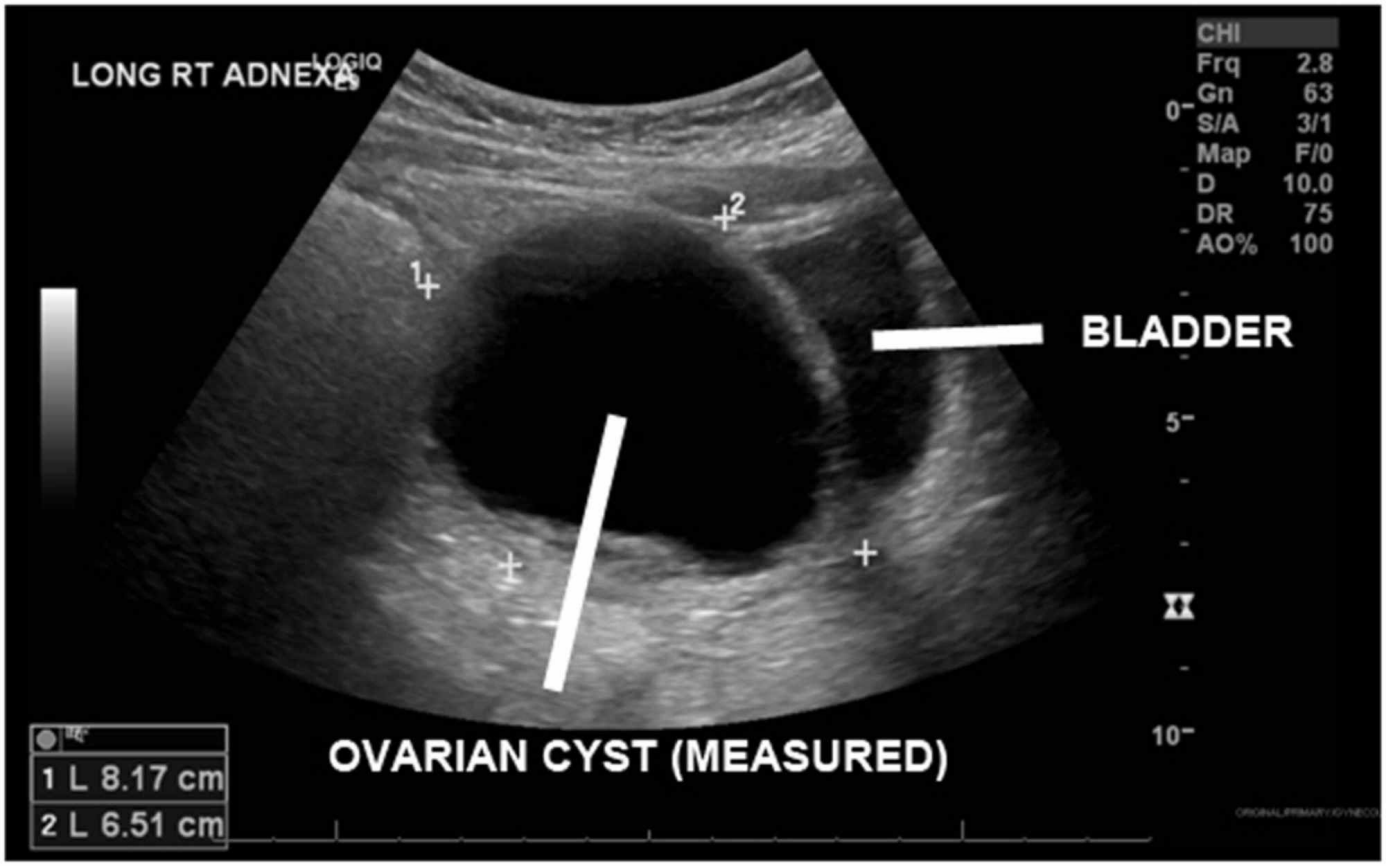
Trans-abdominal pelvic ultrasound (right adnexa)

**Figure 2:**
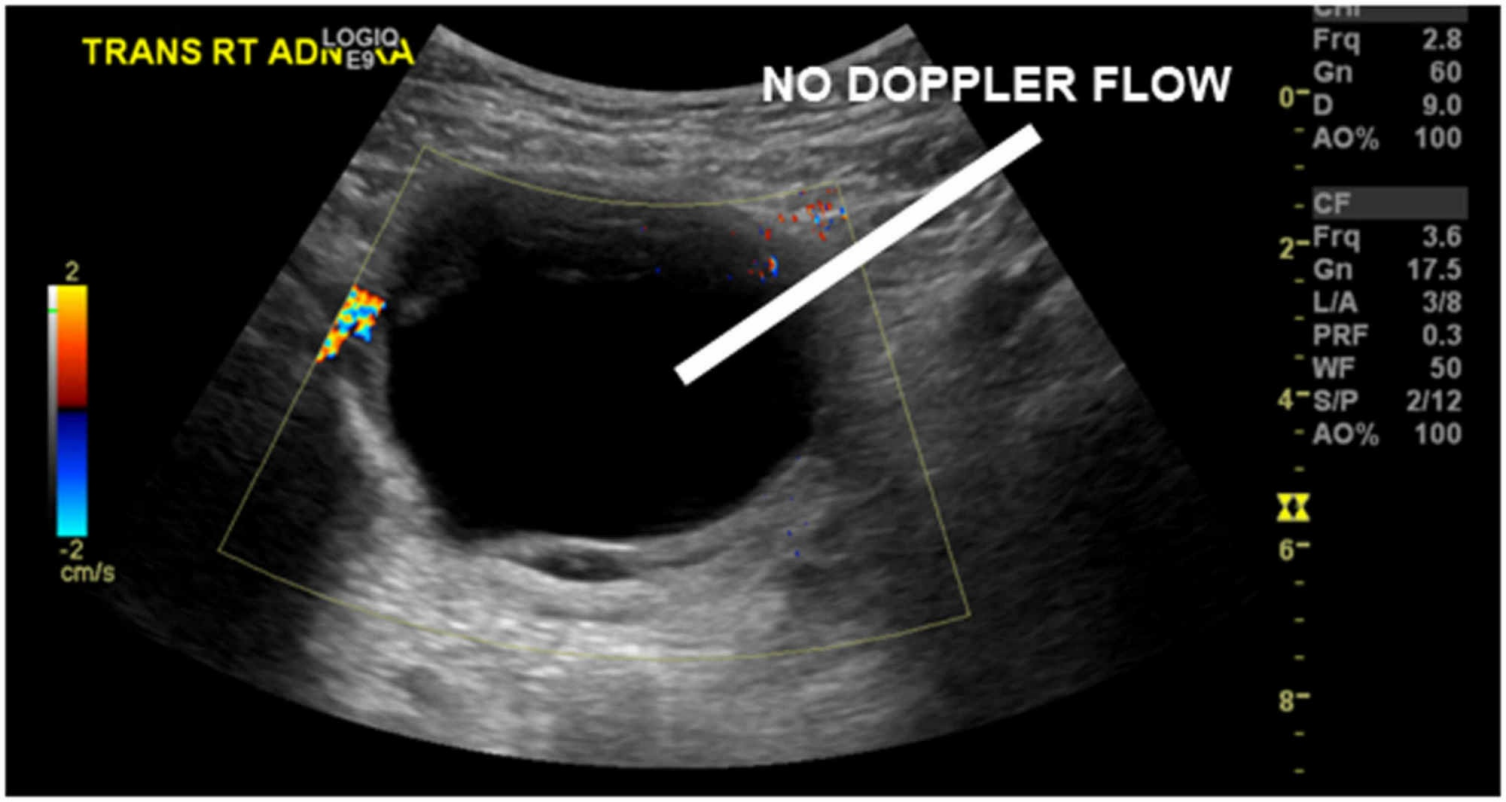
Trans-abdominal pelvic ultrasound (right adnexa with Doppler)

Blood count, chemistry, and urinalysis were completed and unremarkable, and a pregnancy test was negative. Multiple ultrasound views were obtained due to the patient’s larger body habitus, with the requested right lower quadrant ultrasound reporting an inability to visualize the appendix. Subsequently, a trans-abdominal ultrasound of the pelvis reported “Left ovary not visualized. Right ovary w/ large cyst measuring 8.2 x 6.5 x 6.4 cm w/ mural thickening, no Doppler flow visualized.” (Figures 1, 2) Given the patient’s persistent pain and discomfort in the ED, our surgical colleagues ultimately took the patient to the operating room for exploratory laparoscopy without additional cross-sectional CT imaging.

**Figure 3:**
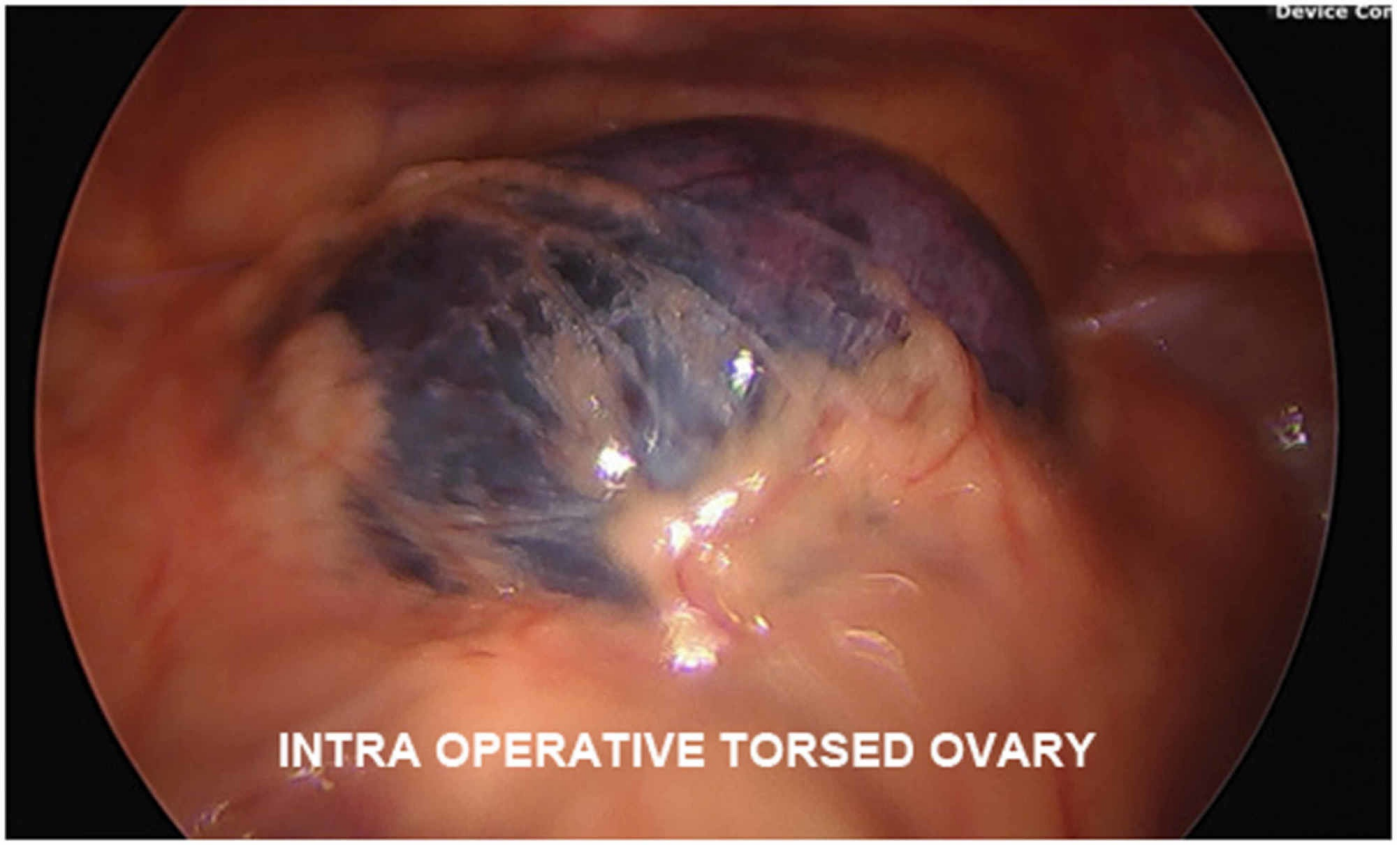
Intra-operative exploratory laparoscopy view of the congested adnexa with a torsed ovary

**Figure 4:**
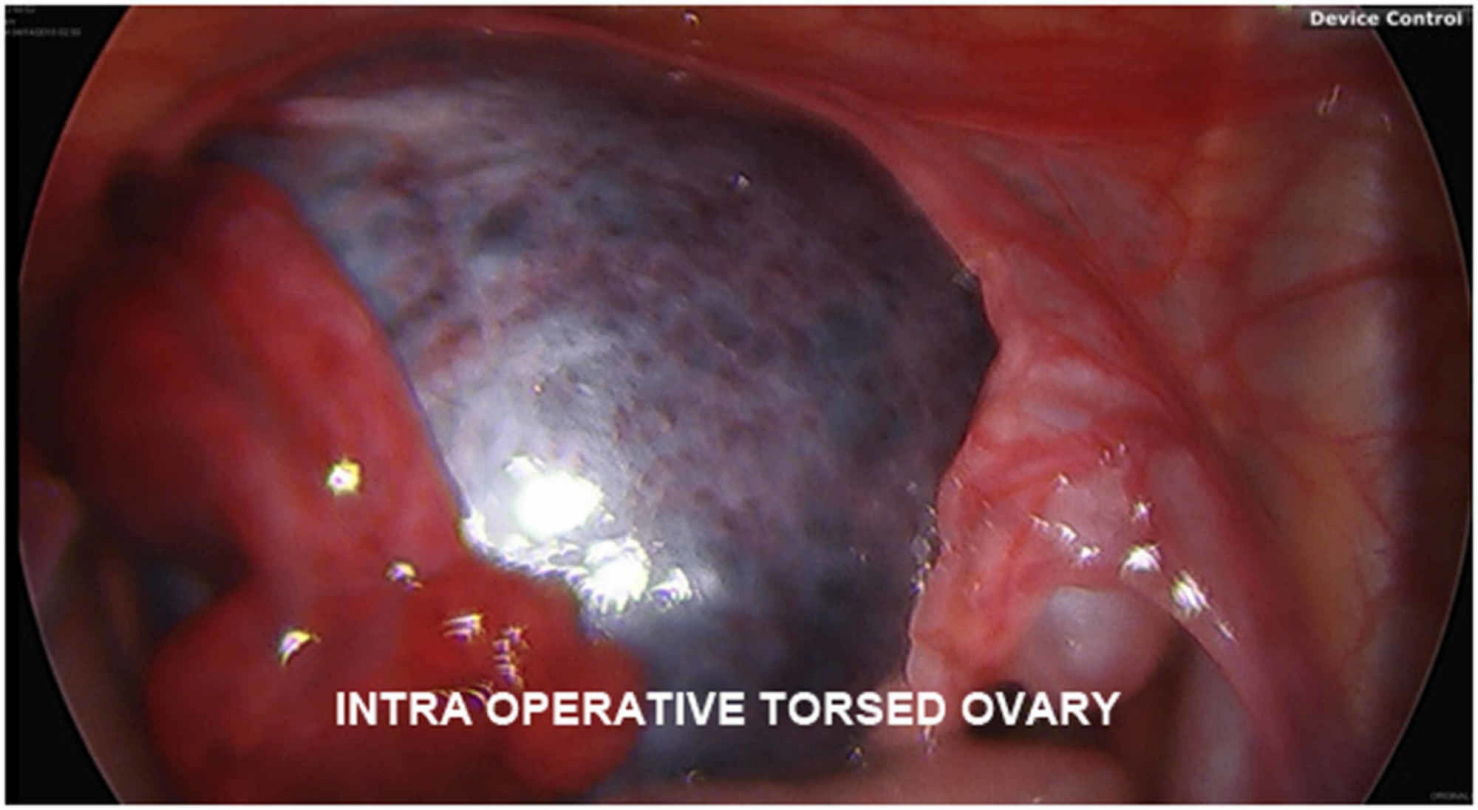
Intra-operative exploratory laparoscopy view of the congested adnexa with a torsed ovary (exposed)

Intra-operatively, a large ovarian cyst with multiple tan-brown, irregularly shaped, soft tissue masses embedded in the cyst wall was extracted from the left adnexa, with a large amount of congested, coagulated blood evacuated as well (Figures 3, 4) The adnexa itself was torsed four times and was unraveled before the cyst was removed. The right ovary was directly visualized and noted to be unremarkable. On pathology several days later, the excised left ovarian cyst was found to be a mature cystic teratoma, with the irregular masses consistent with fully developed teeth.

Surgically, the patient's ovary was able to be successfully salvaged, and she was both pain-free and back to her normal baseline function one month after hospital discharge.

## Discussion

Key differential diagnoses for pediatric patients presenting with vague, non-specific abdominal pain include acute appendicitis, gonadal torsion, volvulus, intussusception, and obstruction. Other common diagnoses include pyelonephritis/cystitis, constipation, and gastritis [2]. For pediatric patients in particular, reported location of pain and even tenderness on examination may be inconsistent and vary with time and repeated evaluation, reminding the physician to maintain a broad differential and re-examine the patient in the ED even if an alternate diagnosis may be more readily available. Non-accidental trauma should be considered if clinical suspicion and concerning history is present.

Ovarian torsion is a rare cause of abdominal pain in girls, accounting for 2.7% of all diagnosed causes of pediatric abdominal pain. An ovarian mass (cyst or neoplasm) is a primary risk factor for torsion; however, up to 52.8% of incidents in one case series demonstrated torsion of a normal ovary/adnexa without malformations [3]. While torsion can occur with a mass of any size or type, larger masses (5 cm in diameter and above) and benign causes are more apt to torse, likely due to their size and malignant masses being more often fixed in place and unable to rotate [3]. Non-malignant causes of ovarian masses may include corpus luteum cysts, cysts related to ovulation induction, ectopic pregnancy, tubo-ovarian abscess, and germ cell tumors [2].

The laterality of the patient’s symptoms, ultrasound results, and confirmed pathology are very unusual in this case. The patient initially complained of left-sided pain, whereas imaging formally reported a right-sided cystic structure and, surgically, the torsion and teratoma were found on the left side. Given the patient’s obesity for age and large cyst size, our suspicion is that the initial ultrasound views of the cystic mass were actually of the left adnexa and displaced so much volume toward the midline that it appeared to exist on the right side of the abdomen, resulting in limited visualization of the left ovary.

Teratomas, or mature dermoid cysts, are the most common type of germ cell tumor, with the majority of them being benign in nature (>95%). While most teratomas are asymptomatic, torsion is a known complication, with rupture and subsequent spillage of contents being relatively uncommon [3-4]. If diagnosed, cystectomy is often suggested to make a definitive diagnosis, prevent potential problems such as torsion, and preserve ovarian tissue, particularly in patients of child-bearing age. Malignant transformation occurs in 0.2-2% of mature dermoid cysts, which complete removal also assists in preventing [3-4].

Both dermoid cysts and cases of ovarian torsion are most prevalent in women in their 20s and 30s and are closely related to reproductive hormonal peaks and cycles. It is important to note that torsion may occur in females of all ages (even fetuses and neonates), including post-menopausal women, particularly if a mass is present [5-6].

The mainstay of treatment is operative, direct visualization of the rotated ovary, tube, or cyst, with an assessment of ovarian viability. Historically, salpingo-oophorectomy was completed due to concern for leaving behind potentially necrotic tissue, whereas the modern surgical approach balances visualization of cystic structures, suspicion of malignancy, and gross appearance, with the likelihood of ovarian conservation with detorsion, particularly in young children [2-3].

## Conclusions

Missing or delaying diagnosis of torsion can result in delayed surgical treatment and preventable loss of reproductive structures, both of which are highly undesirable outcomes from both a clinical and medico-legal perspective. Laterality in both imaging and examination may be inconsistent due to a patient’s age and inability to appropriately verbalize, but it may also be due to the mass effect of pathologic structures altering anatomy and decreasing diagnostic imaging specificity. Ultimately, high clinical suspicion, serial examinations, and early involvement of surgical colleagues can provide the best clinical outcomes for these pediatric patients.
